# Growth hormone remodels the 3D-structure of the mitochondria of inflammatory macrophages and promotes metabolic reprogramming

**DOI:** 10.3389/fimmu.2023.1200259

**Published:** 2023-07-05

**Authors:** Blanca Soler Palacios, Ricardo Villares, Pilar Lucas, José Miguel Rodríguez-Frade, Ana Cayuela, Jonathan G. Piccirillo, Manuel Lombardía, David Delgado Gestoso, Miguel Fernández-García, Cristina Risco, Coral Barbas, Fernando Corrales, Carlos Oscar S. Sorzano, Nuria Martínez-Martín, José Javier Conesa, Francisco J. Iborra, Mario Mellado

**Affiliations:** ^1^ Department of Immunology and Oncology, National Center for Biotechnology/The Spanish National Research Council (CSIC), Madrid, Spain; ^2^ Biocomputing Unit, National Center for Biotechnology/The Spanish National Research Council (CSIC), Madrid, Spain; ^3^ Department of Macromolecular Structures, National Center for Biotechnology/The Spanish National Research Council) (CSIC), Madrid, Spain; ^4^ Functional Proteomics Laboratory, National Center for Biotechnology/The Spanish National Research Council (CSIC), Madrid, Spain; ^5^ Metabolomic and Bioanalysis Center (CEMBIO), Pharmacy Faculty, Universidad San Pablo-CEU, Centre for Universitary Studies (CEU) Universities, Boadilla del Monte, Spain; ^6^ Department of Basic Medical Sciences, Medicine Faculty, Universidad San Pablo-CEU, Centre for Universitary Studies (CEU) Universities, Boadilla del Monte, Spain; ^7^ Tissue and Organ Homeostasis Program, Centro de Biologia Molecular Severo Ochoa, The Spanish National Research Council (CSIC)–Autonomus University of Madrid (UAM), Madrid, Spain; ^8^ Príncipe Felípe Research Centre (Associated Unit to the Biomedicine Institute of Valencia), Biomedicine Institute of Valencia, Valencia, Spain

**Keywords:** growth hormone, macrophages, metabolism, mitochondria, cryo-FIB/SEM

## Abstract

**Introduction:**

Macrophages are a heterogeneous population of innate immune cells that support tissue homeostasis through their involvement in tissue development and repair, and pathogen defense. Emerging data reveal that metabolism may control macrophage polarization and function and, conversely, phenotypic polarization may drive metabolic reprogramming.

**Methods:**

Here we use biochemical analysis, correlative cryogenic fluorescence microscopy and cryo-focused ion-beam scanning electron microscopy.

**Results:**

We demonstrate that growth hormone (GH) reprograms inflammatory GM-CSF-primed monocyte-derived macrophages (GM-MØ) by functioning as a metabolic modulator. We found that exogenous treatment of GM-MØ with recombinant human GH reduced glycolysis and lactate production to levels similar to those found in anti-inflammatory M-MØ. Moreover, GH treatment of GM-MØ augmented mitochondrial volume and altered mitochondrial dynamics, including the remodeling of the inner membrane to increase the density of cristae.

**Conclusions:**

Our data demonstrate that GH likely serves a modulatory role in the metabolism of inflammatory macrophages and suggest that metabolic reprogramming of macrophages should be considered as a new target to intervene in inflammatory diseases.

## Introduction

Macrophages are cells of innate immunity with a high capacity for clearing pathogens and cell debris through phagocytosis, and they also play increasingly defined roles in orchestrating tissue repair. To achieve all this, macrophages display high functional heterogeneity and plasticity. Indeed, depending on their tissue environment and on the activation of specific signaling pathways, they can exhibit pro- or anti-inflammatory functions and phenotypes (1).

Macrophages also display distinct metabolic energy profiles that are linked to their inflammatory status. For example, lipopolysaccharide/interferon (LPS/IFN)-activated M1 macrophages are characterized by enhanced glycolysis (2) and pentose phosphate pathway activity (3), and by suppressed oxidative phosphorylation (OxPhos) efficiency (4), which is suited to the production of pro-inflammatory cytokines, reactive oxygen species (ROS), and nitric oxide (NO). By contrast, IL-4-induced M2 macrophages have an efficient OxPhos system (5) and poor pentose phosphate pathway activity (6), together with enhanced arginase-1 expression and suppressed production of NO and ROS, facilitating the release of anti-inflammatory cytokines, growth factors, and polyamines (7). Furthermore, alveolar macrophages, the most abundant immune cells in the lung in homeostasis, do not rely on glycolysis for LPS-induced inflammation (8), and tumor-associated macrophages modulate their metabolism towards OxPhos and fatty acid oxidation and exhibit functions largely similar to M2 macrophages in a poor glucose environment to preserve their immunosuppressive properties (9). Indeed, in the hypoxic microenvironment of solid tumors, tumor-associated macrophages enhance glycolysis *via* mTOR activation, reducing endothelial glucose availability (10).

Growth hormone (GH) is produced and secreted by somatotrophic cells. Although originally implicated in somatic growth control, GH has numerous functions (11–13) including regulation of the immune system (14–17). Its receptor is expressed by many leukocyte subsets, and GH binding influences the function of B- and T-cells, natural killer cells and macrophages (15). For instance, recombinant human GH (rhGH) administration alters tolerization mechanisms in mice through activation of regulatory T-cells and modulation of Th17 cell plasticity arthritis (18). GH also curtails the development of type I diabetes (19), and contributes to ameliorate symptoms of collagen-induced arthritis (18). In human inflammatory diseases, rhGH administration limits mucosal inflammation in experimental colitis (20), and is protective in patients with active Crohn’s disease (21). In myeloid cells, GH functions as a macrophage-activating factor (22), and stimulates the proliferation of RAW 264.7 macrophages (23). It also plays an important role in granulocyte-macrophage colony-stimulating factor (GM-CSF)-derived macrophage (GM-MØ) reprogramming to an anti-inflammatory and reparative phenotype both *in vitro* and *in vivo* (24). GH treatment of GM-MØ downregulates genes associated with inflammatory macrophages (e.g., *CCR2*, *MMP12*, and *EGLN3*) and upregulates others linked to an M-CSF-polarized macrophage (M-MØ) phenotype (e.g., *HMOX1*, *STAB1*, *IGF1*, and *FOLR2*). It also decreases TNFα production in GM-MØ and increases IL-10 secretion. Contrastingly, no effects of GH are observed in M-MØ (24). Finally, by downregulating the NLRP3 inflammasome in macrophages, GH has been linked to pro-longevity effects that maintain immune system homeostasis during aging (25).

We report here that rhGH treatment of inflammatory macrophages (GM-MØ) *in vitro* reduces glycolysis, lactate and ROS production, and downregulates the expression of important metabolic enzymes including pyruvate kinase M2 (PKM2), pyruvate dehydrogenase kinase 1 (PDK1), ATP-citrate lyase (ACLY), and lactate dehydrogenase A (LDHA). We also show that rhGH treatment of GM-MØ reduces the levels of itaconate, an essential metabolite involved in the immunomodulation of inflammatory macrophages (26). Detailed analysis revealed that rhGH treatment additionally affects the total mitochondria mass of GM-MØ by promoting fusion events and reducing LC3-mediated mitophagy. These findings were validated using confocal live-cell imaging combined with correlative cryogenic fluorescence microscopy and cryo-focused ion-beam scanning electron microscopy (cryo-FIB-SEM). Finally, our results indicated a GH-mediated increase of GM-MØ mitochondrial volume and remodeling of the inner mitochondria membranes, increasing the density of cristae. Overall, the data indicate that GH acts as a metabolic modulator of GM-MØ by modifying the number, mass, dynamics, and internal structure of their mitochondria. Our findings also underscore the importance of cellular metabolism in the coordination of the immune response to environmental conditions and suggest new targets to treat diseases with a high macrophage commitment.

## Materials and methods

### Cell culture

Human peripheral blood mononuclear cells were isolated from buffy coats of healthy donors using Lymphoprep separation (Nycomed Pharma AS, Oslo, Norway), and monocytes were then purified using magnetic cell sorting with CD14 microbeads (Miltenyi Biotech GmbH, Bergisch Gladbach, Germany). Monocytes were cultured at 5 × 10^5^ cells/ml for 7 days at 37°C in a humidified atmosphere with 5% CO_2_ in RPMI medium containing 10% fetal calf serum, and supplemented with GM-CSF (1000 U/ml) or M-CSF (10 ng/ml) (both from ImmunoTools GmbH, Friesoythe, Germany) to generate GM-CSF-polarized (GM-MØ) or M-CSF-polarized (M-MØ) macrophages. Recombinant human GH (Pfizer Genotonorm^®^, 1 μM) was added to 7-day differentiated macrophages for 24 h. When required, 5 × 10^6^ cells were treated with chloroquine (10 ng/ml, 30 min, 37°C; Sigma-Aldrich, Madrid, Spain), and 1 × 10^6^ cells were treated with rapamycin (10 ng/ml, 30 min, 37°C; Calbiochem, San Diego, CA) prior to treatment with rhGH.

### Gene-set enrichment analysis

RNA-sequencing data have been deposited in the Sequence Read Archive (SRA) (https://www.ncbi.nlm.nih.gov/sra) under accession no. PRJNA555143. Gene-set enrichment analysis (GSEA) was performed using the gene-sets in “HALLMARK” human collection (27) available at http://software.broadinstitute.org/gsea/index.jsp.

### Immunohistochemistry and multicolor confocal microscopy

The following antibodies were used: anti-MFN2 (12186-1-AP, Proteintech, Rosemount, IL), anti-PKM2 (sc365684; Santa Cruz Biotechnology, Santa Cruz, CA), anti-LDHA (2012; Cell Signaling Technology, Danvers, MA), anti-ACLY (ab407793; Abcam, Cambridge, UK), anti-SDH (CL0349; ThermoFisher Scientific, Waltham, MA), anti-IDH (8H26L4; ThermoFisher Scientific), anti-TFAM (16832595; ThermoFisher Scientific), anti-PPARGC1A (NBP1-04676; Bio-techne, Minneapolis, MN), and isotype-matched control antibodies and fluorochrome-conjugated secondary antibodies (Jackson ImmunoResearch Laboratories, West Grove, PA). Macrophages were plated on poly-L-lysine (Sigma-Aldrich)-coated coverslips, fixed with 4% formaldehyde and, when indicated, permeabilized with 0.1% saponin for 10 min. Cells were then blocked for 10 min with 1% human immunoglobulins before incubation with primary (1–5 μg/ml) and appropriate secondary antibodies.

For the quantitative analysis of mitochondria, macrophages were incubated in complete RPMI containing 100 nM MitoTracker FITC (11589106; Invitrogen, Carlsbad, CA) and CMXRos-Red (M7512; ThermoFisher Scientific) for 30 min, and then with 2 μg/ml DAPI (Sigma-Aldrich) for an additional 2 min. Cells were washed twice and resuspended in PBS-formaldehyde (2%). For cell imaging, macrophages seeded on confocal dishes were placed in a micro-incubator system at 37°C and 5% CO_2_ in a humidified environment. Imaging was performed with an inverted confocal microscope (SP5; Leica Microsystems, Buffalo Grove, IL), using the 20 PL-APO NA 0.7 (dry) and the 63 PL-APO NA 1.3 (glycerol immersion) objectives.

Quantification of protein expression was performed using similar acquisition settings in all cells and localized with DAPI and differential interference contrast. Mean fluorescence intensities (arbitrary units) within regions of interest (ROIs) were assessed using ImageJ (NIH, Bethesda, MD) (28). At least six independent samples were evaluated for each type of macrophage. After background subtraction, data were plotted using Prism software (GraphPad Software, La Jolla, CA).

### Lactate quantitation

Supernatants were analyzed for the presence of lactate using the Lactate Colorimetric Assay Kit II (#K627-100; Quimigen, Madrid, Spain).

### Detection of intracellular ROS

Cells were incubated with 20 μM H_2_DCF-DA (ab113851; Abcam) in culture medium for 30 min at 37°C and 10% CO_2_. H_2_DCF-DA-loaded cells were then washed, resuspended in RPMI, and further incubated for 30 min. Cell fluorescence was quantified using flow cytometry (FACS Aria, BD Biosciences, San Jose, CA).

### Detection of mitochondrial ROS

Cells were incubated with MitoROS 580 (25169-500; Cayman Chemical, Ann Arbor, MI) for 30 min, and then with 2 mg/ml DAPI (Sigma-Aldrich) for an additional 2 min. After incubation, cells were washed twice and resuspended in PBS-formaldehyde (2%). For cell imaging, macrophages seeded on confocal dishes were placed in a micro-incubator system of 37°C and 5% CO_2_ in a humidified environment. Imaging was performed with an inverted confocal microscope (SP5; Leica Microsystems) using the 20 PL-APO NA 0.7 (dry) and the 63 PL-APO NA 1.3 (glycerol immersion) objectives, as described above. Quantification of protein expression was performed as described in the immunohistochemistry and multicolor confocal microscopy section.

### Gas chromatography–mass spectrometry analysis of tricarboxylic acid metabolites

Frozen pellets (∼1.5 × 10^7^ cells) were thawed in an ice bath (0°C) with 600 μl of MeOH:H_2_O (50:50, *v/v*) and were then sonicated (16 pulses, 0.5 s pulse length, 80% amplitude). Subsequently, 250 μl of the lysed suspension was quickly mixed with 350 μl of cold (-20°C) MeOH containing 25 mg/l of d31-palmitic acid (internal standard 1). The resulting mixture was vortex-mixed (t = 1 h) and centrifuged (4000 × *g*, T = 20 °C, t = 60 min). Supernatants were dried under high vacuum. Sample extracts then underwent a two-step derivatization procedure described for the determination of polar metabolites by gas chromatography–mass spectrometry (29). Derivatized sample extracts were vortex-mixed (t = 2 min), ultrasonicated in a bath sonicator (t = 5 min) cooled to room temperature (t = 30 min) and then mixed with 20 mg/l tricosane (IS 2) in heptane (Panreac, Castellar del Vallés, Spain). Sample extracts were injected and analyzed in an Agilent 7980B GC system coupled to an Agilent 7250 QTOF/MS analyzer (Agilent Technologies, Waldbronn, Germany) using a previously-described method (29). Helium carrier gas flow rate was 0.76 ml/min. Column temperature was initially held at 60°C for 1 min, then increased at 10°C/min until 325 °C, which was maintained for 10 min. Full-MS was selected as acquisition mode, with an *m/z* range of 50–600 and an acquisition time of 200 ms. Sample extracts were analyzed in a randomized order.

MS traces corresponding to tricarboxylic acid (TCA) cycle intermediates were extracted and integrated using Agilent MassHunter Quantitative Analysis (v. 10.2, build 10.2.733.8, Agilent Technologies, Waldbronn, Germany). Abundances were subjected to blank subtraction and subsequent internal standard normalization. The identity of the measured compounds (citrate, isocitrate, *trans*-aconitate, *cis*-aconitate, fumarate and malate) was confirmed by spectral and retention times comparison of MS-grade pure standards (all from Sigma-Aldrich, Steinheim, Germany) with metabolite signals obtained in samples.

### Determination of intracellular itaconate levels

Intracellular itaconic acid levels were measured using high performance liquid chromatography. Pellets from 2 × 10^7^ macrophages were resuspended in PBS, pH 7.4 and lysed by three freeze-thaw cycles. Samples were bath-sonicated (5 min, room temperature) in two steps with an intermediate freeze step. After centrifugation (17,000 × g, 15 min, 4°C), debris-free lysates were transferred to new tubes. Proteins were then precipitated using 100 mM hydrochloric acid and centrifuged (17,000 × g, 15 min, 4°C). Remaining supernatants were injected into a column of 250 × 2 mm (Col Kromophase 100 C18 5.0 mm; Scharlau Chemie SA, Barcelona, Spain) and itaconate was quantified using UV detection at 210 mm. A standard curve (see **Supplementary Figure 1A**) was obtained using pure itaconic acid (0.50–500 μM) (I29204; Sigma-Aldrich). Results were normalized using the protein concentration of each sample.

### Quantitative real-time PCR

Total RNA from cells was extracted using the Nucleospin RNA/Protein kit (Macherey-Nagel, Düren, Germany). RNA was retrotranscribed using SuperScript III reverse transcriptase (Invitrogen). For mitochondrial DNA quantitation, 3 × 10^6^ cells were lysed in 50 mM Tris·HCl, 5 mM EDTA, 100 mM NaCl, 1% SDS, 100 μg/ml proteinase K and incubated for 2 h at 56°C. DNA was precipitated with isopropyl alcohol, washed with 70% ethanol and resuspended in water. qPCR analyses was performed with the GoTaq qPCR Master Mix (Promega, Madison, WI). Samples were quantified using the QuantStudio 5 software (Applied Biosystems). Oligonucleotides for selected genes were designed employing the Roche Universal ProbeLibrary Assay Design Center and (30) (**Table 1**). Data from triplicate samples were averaged and normalized using the 2^-ΔCt^ comparative threshold method, according to the expression levels of the housekeeping gene *TBP* or, for mitochondrial DNA quantification, to the average levels of nuclear *APP* and *B2M* DNA.

**Table 1:** List of primer couples generated for qPCR.

Gene	Forward	Reverse
*CS*	5’-GGGGCCATTGACTCTAACCT-3’	5’-TACATTGCCACCCTCATGG-3’
*TFAM*	5’-GAACAACTACCCATATTTAAAGCTCA-3’	5’-GAATCAGGAAGTTCCCTCCA-3’
*GLUT1*	5’-CCTGCAGTTTGGCTACAACAC-3’	5’-GAGGATGCTCTCCCCATAGC-3’
*LDHA*	5’-AGGCCCGTTTGAAGAAGAGT-3’	5’-CACTTACAAGCCAAACCAACCAA-3’
*IRG1*	5’-GCTTCCTACAGTTGGCTGCT-3’	5’-ATGTGCAGGAAAACGCTTAAA-3’
*MFN1*	5’-CAGCAAAGGAAGTTCTTAGTGC-3’	5’-GCTGACTGCGAGATACACTC-3’
*MFN2*	5’-CCATGAGGCCTTTCTCCTTAC-3’	5’-GTGGGCACTTAGAGTTGGG-3’
*SLC25A1*	5’-AAGTTCATCCACGACCAGAC-3’	5’-GTTCGAGCCCTGCTTCAG-3’
*PPARGC1A*	5’-TGTGGAGACAGGGGCTTTTA-3’	5’-CTTGGGGTCATTTGGTGACT-3’
*mtCO2*	5’-ACGCATCCTTTACATAACAGAC-3’	5’-GCCAATTGATTTGATGGTAAGG-3’
*mtRNR2*	5’-AACTCGGCAAATCTTACCC-3’	5’-AATACTGGTGATGCTAGAGGTG-3’
*mtATP6*	5’-TCCCTCTACACTTATCATCTTCAC-3’	5’-GACAGCGATTTCTAGGATAGTC-3’
*APP*	5’-TTTTTGTGTGCTCTCCCAGGTCT-3’	5’-TGGTCACTGGTTGGTTGGC-3’
*ß2M*	5’-TGCTGTCTCCATGTTTGATGTATCT-3’	5’-TCTCTGCTCCCCACCTCTAAGT-3’

### Fusion-fission experiments and image analysis

Macrophages were plated on poly-L-lysine (Sigma-Aldrich)-coated coverslips, fixed with 4% formaldehyde and, when indicated, permeabilized with 0.1% saponin for 10 min. Cells were then blocked for 10 min with 1% human immunoglobulins, and incubated with anti-TOM22 (1 μg/ml, Sigma-Merck; HPA003037) and appropriate secondary antibodies. Imaging was acquired with an inverted confocal microscope (YODA WF/TIRFM, Leica Microsystems), using the 20 PL-APO NA 0.7 and the 63 PL-APO NA 1.3 objectives and an EM-CCD camera (Andor DU 885-CS0-#10-VP; Andor Technology, Belfast, UK).

The approach to quantitatively evaluate the mitochondrial characteristics is summarized in **Supplementary Figures 2A, B**. Analysis of mitochondrial morphology was performed using the macro complement Mitochondrial Flow of ImageJ (https://github.com/QuantitativeImageAnalysisUnitCNB/MitochondrialFlow_) (31). Briefly, images were first cropped to show a single cell per image. Next, selected cells were processed using an enhanced local contrast (CLAHE macro) and a binary image was obtained and processed using as threshold (Size = 0.06 μm^2^-Infinity, Circularity = 0.00 -1.00), to finally obtain the Area, Perimeter, and Shape Descriptor values. The Form Factor (FF) data were calculated as the inverse of the “Circularity” output value. The network connectivity analysis was performed using the Skeletonize 2D to generate a skeleton map, and the Analyze Skeleton plugin to calculate the number of branches, branch lengths, and branch junctions.

### Western blotting

Cell lysates were obtained in RIPA buffer containing 2 mM Pefabloc (Merck, Darmstadt, Germany), 2 mg/ml aprotinin/antipain/leupeptin/pepstatin, 10 mM NaF, and 1 mM Na_3_VO_4_. Cell lysates (30 μg/ml) was subjected to SDS-PAGE and transferred onto Immobilon polyvinylidene difluoride membranes (Millipore, Billerica, MA). Protein detection was carried out using anti-OPA1 (#80471; Cell Signaling Technology), anti-human LC3-I/II (#12741; Cell Signaling Technology), anti-HMGB1 (110117; HMGBiotech, Milan, Italy), and anti-PDK1 (#3062; Cell Signaling Technology) antibodies. Densitometric analysis of the blots was performed using Image Quant software (Amersham Bioscience, Buckinghamshire, UK). For loading control, membranes were reblotted with anti-vinculin monoclonal antibody (#700062, Sigma-Aldrich).

### Correlative cryogenic fluorescence microscopy and cryo FIB-SEM

#### Vitrification

Cells (5 × 10^5^) were seeded on Quantifoil silicon oxide grids R 1/4 finder F1 Au grids, 200 mesh (Quantifoil Micro Tools GmbH, Jena, Germany). Cells were then cultured in RPMI medium (24 h, 37°C, 5% CO_2_) and stained with MitoTracker Red FM (M22425; ThermoFisher Scientific) for 30 min at 37°C. Grids were vitrified by plunge-freezing using a Leica EM GP2 grid plunger (Leica Microsystems, Vienna, Austria) set to 37°C, 95% humidity and a blotting time of 7 s by the grid side opposite to the growing cells. Immediately before vitrification, 3 μl of Dynabeads MyOne Carboxylic Acid (1 μm; ThermoFisher Scientific) were added at a concentration of 0.5 mg/ml to each of the samples. Vitrified grids were mounted under liquid nitrogen in c-clip rings (ThermoFisher Scientific).

### Image acquisition

#### Cryo-fluorescence microscopy

The approach to quantitate the mitochondrial characteristics is summarized in **Supplementary Figure 3**. Vitrified samples were analyzed by cryo-fluorescence microscopy using a LSM 900 confocal microscope (Carl Zeiss NTS GmbH, Oberkochen, Germany) equipped with a Linkam CSM196 cryo-stage (Linkam Scientific Instruments, Cambridge, UK). Fluorescence images were acquired with an LD A-Plan 20×/0.35 Ph1 objective using two channels, brightfield and red fluorescence emission, both with a pinhole aperture of 67 μm. Brightfield images were collected using an ESID detector (photodiode) with an excitation light of 400 nm. Red fluorescence images were collected using a GaAsP-PMT detector and an ex/em wavelength of 578/598 nm. Whole grids were imaged to generate a map of stitched images (9 images per grid) to screen grids and locate cellular coordinates of interest.

#### Cryo-FIB-SEM volume imaging

Cryo-fluorescence microscopy samples were transferred to the cryo-FIB-SEM microscope in liquid nitrogen using a Leica EM-VCM500 machine and a cryo-holder suitable for c-clipped grids (Leica Microsystems, Vienna, Austria). Prior to sample loading into the FIB-SEM microscope, grids were metalized by platinum sputtering (4 nm) using a Leica ACE600 cryo-sputter coater equipped with a cryo-stage (Leica Microsystems). Metallized samples were then transferred to a pre-cooled Crossbeam 550 FIB-SEM microscope (Carl Zeiss) equipped with a cryo-stage (Leica Microsystems) using an EM-VCT100 shuttle (Leica Microsystems). The cryo-stage was positioned at a working distance of 5.1 mm and tilted to 11° and further protected by a layer of cold-deposited organic-platinum (3 depositions in cycles of 30 s not heating the source at a working distance of 8 mm). Serial sectioning of the sample was followed to generate the FIB-SEM volumes using SmartFIB software (Carl Zeiss). Imaging conditions were 1.8 kV acceleration voltage and a probe current of 36 pA using the InLens detector and at a final pixel size between 4 and 9 nm. FIB milling was done at 30 kV and 300 pA with a sectioning step between 20 and 25 nm. Four different cells from each condition were imaged.

### Image processing and segmentation

Raw FIB-SEM image stacks pre-processing was done using ImageJ. To remove the curtain artefact in the raw images a vertical band-pass filter between 1 and 300 pixels was applied. Image stack alignment was done using Linear Stack Alignment with SIFT (32). Aligned stacks were then submitted to semi-manual segmentation and statistical analysis using Amira software (ThermoFisher Scientific) (**Video 1**). We obtained the morphological data of the volume and elongation of each mitochondrion in untreated or rhGH-treated macrophages. Elongation was defined as the ratio of the medium and the largest eigenvalue of the covariance matrix calculated for each mitochondrion. To evaluate fusion-fission events in 3D we used ImageJ. The approach for mitochondrial characteristics quantitation and subsequent morphology analysis was obtained using a public Java-based “MitochondrialAnalyzer” plugin operational under ImageJ software (https://github.com/QuantitativeImageAnalysisUnitCNB/MitochondrialAnalyzer_).

### Statistical analysis

For comparison of mean or median, and unless otherwise indicated, statistical significance of the data was evaluated using a paired *t*-test. With the exception of GSEA, a p-value <0.05 was considered significant (*p<0.05, **p<0.01, ***p<0.001, ****p<0.0001). Statistical parameters used in the GSEA analysis were as previously described (33, 34); here, a nominal p-value <0.001 with False Discovery Rate Q (FDRq) value <0.01 was considered significant.

## Results

### Growth hormone downregulates the expression of key enzymes involved in glucose metabolism in human GM-MØ

It was previously demonstrated that the oxygen consumption rate (OCR) and aerobic glycolysis (measured as the extracellular acidification rate [ECAR]) are higher in GM-MØ than in M-CSF-derived macrophages (M-MØ), concomitant with the elevated expression of genes encoding glycolytic enzymes (35). This suggests a potentially important role for metabolic reprogramming in macrophage polarization and function.

**Figure 1:**
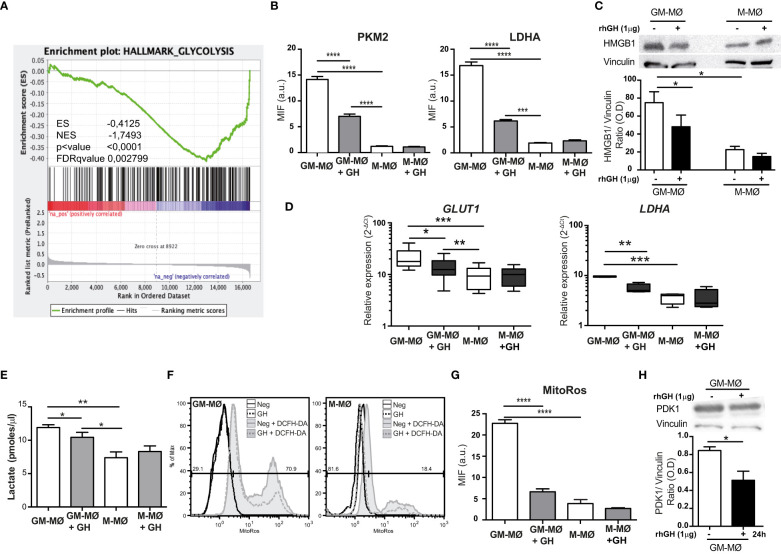
rhGH decreases the expression of specific glycolysis enzymes and reduces lactate and ROS production in GM-MØ. **(A)** GSEA on the ‘log fold-change-ranked’ list of genes obtained from untreated versus rhGH-treated GM-MØ according to limma analysis, using the pre-defined glycolysis genes set (Glycolysis Hallmark; https://www.gsea-msigdb.org/gsea/msigdb/human/geneset/HALLMARK_GLYCOLYSIS.html). Vertical black lines indicate the position of each of the genes comprising the glycolysis gene-set. The normalized enrichment score (NES), nominal p value and False Discovery Rate-adjusted Q value (FDRq) are presented. **(B)** Quantitative analysis of confocal images of GM-MØ and M-MØ treated or not with rhGH (1 μg/ml, 24 h), stained with anti-PKM2, or -LDHA monoclonal antibodies. Equatorial-plane images from 200–300 cells were collected in random fields and analyzed using ImageJ. Results are shown as mean ± SD after background subtraction (mean fluorescence intensity, MFI) (n=8). Paired *t-*test ***p<0.001, ****p<0.0001. **(C)** GM-MØ and M-MØ treated or not with rhGH (1 μg/ml, 24 h) were lysed and the expression of HMGB1 was determined by western blotting using an anti-HMGB1 monoclonal antibody. As loading control membranes were reblotted with an anti-vinculin monoclonal antibody. A representative experiment of 5 performed is shown. Densitometric analysis of the western blotting performed is also shown. Results were normalized to that of vinculin. Results are shown as mean ± SEM (n=5). Paired *t-*test, *p<0.05. **(D)** Relative expression levels of *GLUT1* and *LDHA* in GM-MØ and M-MØ untreated or treated with rhGH (1 μg/ml, 24 h). Results are shown as 2^−ΔCt^ relative to the mean of internal TBP expression and correspond to triplicate determinations (n=12); box and whisker plots represent the median, second and third quartiles, and the minimum to maximum values. Paired *t-*test *p<0.05, **p<0.01, ***p<0.001. **(E)** Lactate determination in culture supernatants of GM-MØ and M-MØ untreated or treated with rhGH (1 μg/ml, 24 h). Results are shown as mean ± SD (n=4). Paired *t-*test *p<0.05, **p<0.01. **(F)** ROS levels in GM-MØ and M-MØ untreated or treated with rhGH (1 μg/ml, 24 h) determined by incubation with DCFH-DA for 60 min followed by flow cytometry analysis. Representative plots are shown (n=8). **(G)** Quantitative analysis of confocal images of GM-MØ and M-MØ untreated or treated with rhGH (1 μg/ml, 24 h) and stained with MitoROS, data were processed as in **(B)** (n=8). Results are shown as mean ± SD (n=4). Paired *t*-test ****p<0.0001. **(H)** GM-MØ treated or not with rhGH (1 μg/ml, 24 h) were lysed and the expression of PDK1 was determined by western blotting using an anti-PDK1 monoclonal antibody. As a loading control, membranes were reblotted with an anti-vinculin monoclonal antibody. A representative experiment of 5 performed is shown. Densitometric analysis of the western blotting performed is also shown. Results are shown as mean ± SEM (n=5). Paired *t-*test, *p<0.05.

We recently reported that GH-stimulated GM-MØ have an anti-inflammatory and reparative profile (24), and we hypothesized that, under such experimental conditions, GH might influence inflammatory macrophage metabolism and reprogramming. To test this, we first performed a GSEA of RNA-sequencing data from GH-treated GM-MØ (24). Using an RNA expression fold-change (FC= log_2_ (GM-MØ/GM-MØ+GH)) FC=1 as a cut-off, the human Hallmark collection of gene-sets rendered no positively regulated gene-sets but six negatively regulated gene-sets (p-value <0.001, FDRq <0.01). Two of them, Glycolysis (genes encoding proteins involved in glycolysis and gluconeogenesis) and MTORC1 (genes up-regulated through mTORC1 complex activation), corresponded to lower metabolic activities as compared with the untreated cells (**Figure 1A**; **Supplementary Figure 1B**).

We next questioned whether the GSEA data correlated with differences in the expression of key enzymes in aerobic glycolysis. We first examined the expression of PKM2 and LDHA in untreated or GH-treated GM-MØ by immunostaining (**Supplementary Figure 1C**). Untreated M-MØ were also included as a control. Quantification of the mean fluorescence levels of the images revealed that the expression of both proteins was significantly lower in GH-treated GM-MØ than in untreated cells (**Figure 1B**). PKM2 activity is associated with the cellular secretion of TNFα and HMGB1. GH treatment of GM-MØ reduces TNFα secretion (24), and we also noted that HMGB1 levels were lower in GM-MØ than in M-MØ, and HMGB1 expression was also significantly reduced in GH-treated GM-MØ (**Figure 1C**). RT-q-PCR analysis of the same cells demonstrated that the gene expression levels of the glucose transporter *GLUT1* and *LDHA* were significantly lower after GH treatment (**Figure 1D**). These findings suggest that GH drives pyruvate to the mitochondria, which would increase the flux of acetyl-CoA through the TCA cycle and also increase the production of NADH and subsequent O_2_ consumption as the final electron acceptor in the electron transport chain. Supporting this, we detected a significant decrease in lactate accumulation (**Figure 1E**) and cytosolic ROS production (**Figure 1F**; **Supplementary Figure 1D**) which correlated with a reduction of mitochondrial ROS (**Figure 1G**; **Supplementary Figure 1C**), in GM-MØ treated with GH. Pyruvate dehydrogenase kinase1 (PDK1) is a key regulatory enzyme for glucose metabolism, and reduced PDK1 activity shifts cell metabolism from aerobic glycolysis towards OxPhos (36). We thus evaluated the effect of GH treatment on PDK1 expression, finding that it was significantly lower in GH-treated GM-MØ than in untreated cells (**Figure 1H**). Altogether, these data indicate that GH tempers aerobic glycolysis in GM-MØ, a metabolic pathway associated with inflammatory macrophages (5).

**Figure 2:**
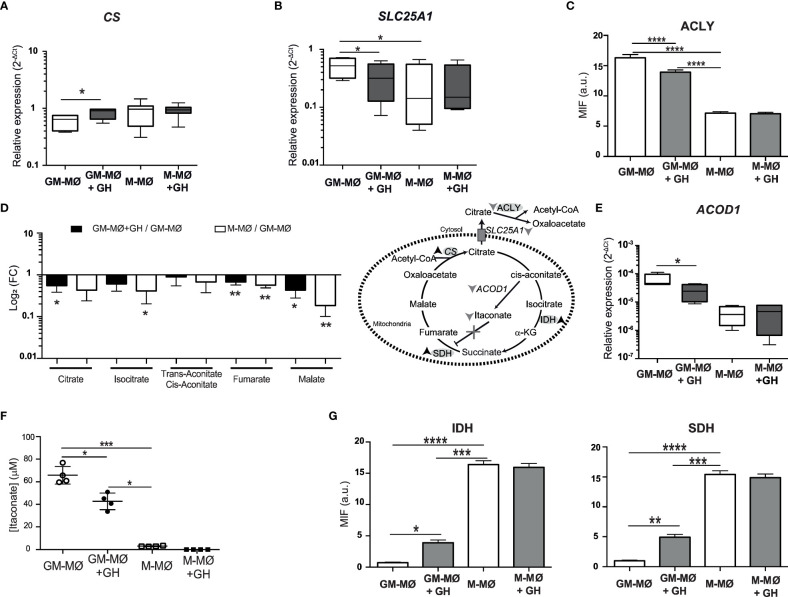
rhGH modulates TCA metabolism in GM-MØ. *CS* **(A)** and *SLC25A1* **(B)** mRNA expression levels determined by RT-qPCR in GM-MØ and M-MØ untreated or treated with rhGH (1 μg/ml, 24 h). Results are shown as 2^−ΔCt^ relative to the mean of internal *TBP* expression and correspond to triplicate determinations (n=8); box and whisker plots represent the median, second and third quartiles, and the minimum to maximum values. Paired *t-*test *p<0.05. **(C)** Quantitative analysis of confocal images of GM-MØ and M-MØ untreated or treated with rhGH (1 μg/ml, 24 h), stained with an anti-ACLY monoclonal antibody. Equatorial-plane images from 200–300 cells were collected in random fields and analyzed using ImageJ. Results are shown as mean ± SD after background subtraction (mean fluorescence intensity, MFI) (n=8). Paired *t*-test, ****p<0.0001. **(D)** Abundance of metabolites in rhGH-treated GM-MØ and M-MØ relative to GM-MØ. Data presented as log_2_ of fold-change (FC) mean ± SD of three independent samples. Paired *t*-test, *p<0.05, **p<0.01. Effects of rhGH treatment on enzymes and products of the TCA cycle. Down arrows indicate downregulation, up arrows indicate upregulation and X represents blockade. **(E)** *ACOD1* mRNA expression levels determined by RT-qPCR in GM-MØ and M-MØ untreated or treated with rhGH 1 (μg/ml, 24 h). Results are shown as in **(A, B)**. Paired *t-*test *p<0.05. **(F)** Intracellular levels (n=4 cultures) of itaconate in GM-MØ and M-MØ untreated or treated with GH (1 μg/ml, 24 h). All plots represent mean ± s.e.m. (n=8) Paired *t-*test *p<0.05, ***p<0.001. **(G)** Quantitative analysis of confocal images of GM-MØ and M-MØ treated or not with rhGH (1 μg/ml, 24 h), stained with anti-IDH and -SDH monoclonal antibodies. Equatorial-plane images from 200–300 cells were collected in random fields and analyzed using ImageJ software. Results are shown as mean ± SD after background-subtraction. Paired *t*-test *p<0.05, **p<0.01, ***p<0.001, ****p<0.0001.

The TCA cycle is important in cell metabolism and begins with the generation of citrate, which is formed by the condensation of oxaloacetate with acetyl-CoA generated from fatty acids, amino acids, or pyruvate oxidation. We found that GH treatment significantly increased the gene expression levels of *CS* (citrate synthase) in GM-MØ but not in M-MØ (**Figure 2A**). Citrate is transported from mitochondria to the cytosol by the action of SCL25A1, where it is used for acetylation reactions. We observed that GH treatment of GM-MØ downregulated *SCL25A1* mRNA levels (**Figure 2B**). We also noted that GH decreased the expression of ACLY in GM-MØ (**Figure 2C**; **Supplementary Figure 1C**). Because these data suggest an effect of GH on the TCA cycle, we next evaluated several TCA metabolites (citrate, isocitrate, trans-aconitate-cis-aconitate, fumarate and malate), finding higher levels of all in GM-MØ than in M-MØ and a fall in levels following GH treatment of GM-MØ (**Figure 2D**). Citrate can also be converted to *cis*-aconitate, a metabolic intermediate of itaconate and succinate, by the action of aconitase. We observed that GH treatment reduced the expression of *ACOD1* (aconitate decarboxylase 1), which is involved in itaconate production, in GM-MØ but not in M-MØ (**Figure 2E**), concomitant with a decrease in itaconate levels (**Figure 2F**). These results agree with the evident increase in IDH2 (isocitrate dehydrogenase (NADP (+)) 2) and SDH (succinate dehydrogenase) protein levels in GM-MØ treated with GH (**Figure 2G**; **Supplementary Figure 1C**).

### Growth hormone affects the total number of mitochondria and their morphology and dynamics in GM-MØ

**Figure 3:**
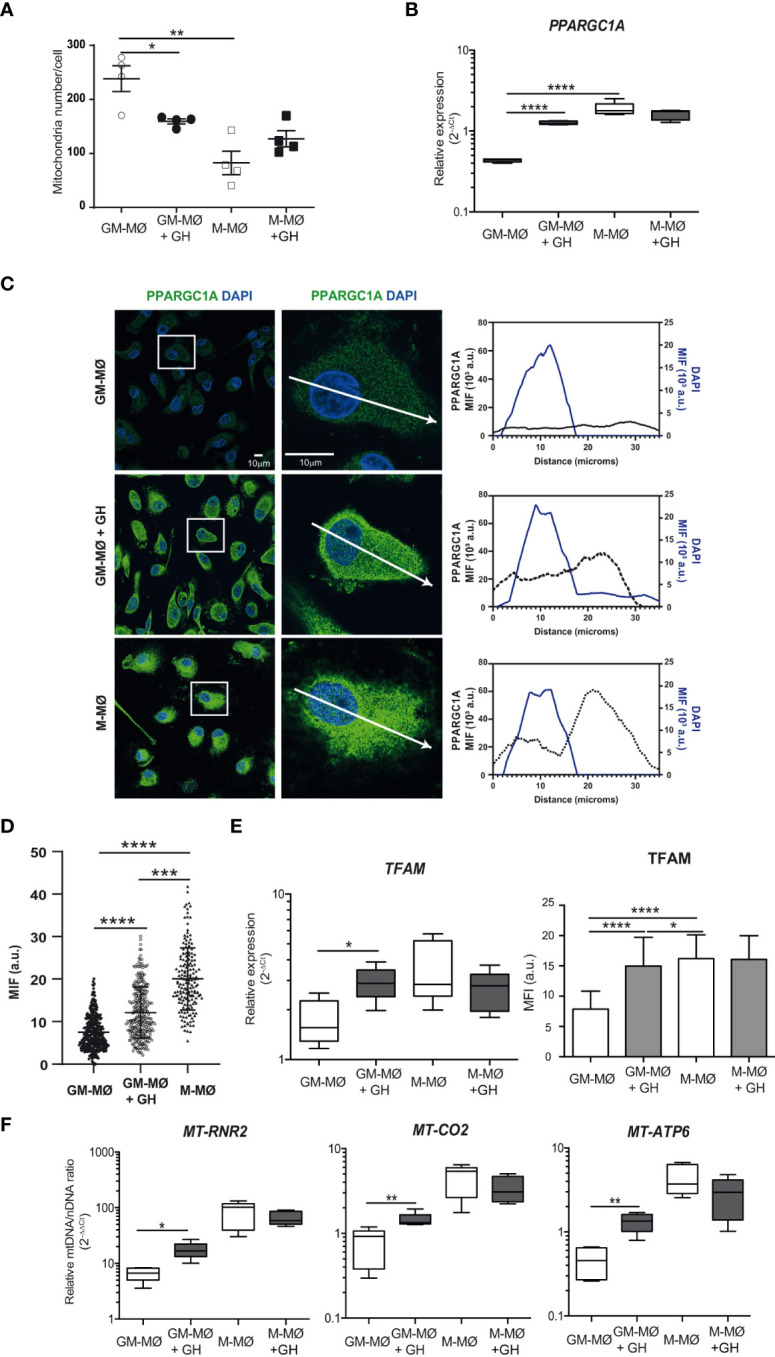
rhGH modulates mitochondrial number and mtDNA abundance in GM-MØ **(A)** Quantitative analysis of mitochondria number/cell on confocal microscopy images of mitochondria using MitoTracker Green. Data correspond to triplicate determinations (n = 8); dot plots represent median, and interquartiles ranges. Paired *t-*test *p<0.05, **p<0.01. **(B)** *PPARGC1α* mRNA expression levels determined by RT-qPCR in GM-MØ and M-MØ untreated or treated with rhGH (1 μg/ml, 24 h). Results are shown as 2^−ΔCt^ relative to the mean of internal *TBP* expression and correspond to triplicate determinations (n=8); box and whisker plots represent the median, second and third quartiles, and the minimum to maximum values. Paired *t*-test ****p<0.0001. **(C)** Equatorial-plane images of untreated or rhGH-treated GM-MØ and M-MØ cells as indicated, stained with anti-PPARGC1α monoclonal antibody followed by a secondary antibody coupled to Alexa Fluor 488 (green), and DAPI (cyan) and analyzed using ImageJ. Scale bar, 10 μm. Zoom images (2,5x) of representative cells. Profiles of relative mean fluorescence distribution of PPARGC1α (green) and DAPI (cyan) at a equatorial-plane of the cells (arrow in the zoom image). **(D)** Total PPARGC1a fluorescence values at the nucleus of cells in **(C)** represented as mean ± SD after background subtraction (mean fluorescence intensity, MFI), each dot represents a single cell. Data correspond to a representative experiment (n = 3). Statistical analysis, two-tailed unpaired Student t test was applied (Prism 4.0; GraphPad). ***p<0.001; ****p<0.0001. **(E)** Quantitative analysis of confocal images of GM-MØ and M-MØ treated or not with rhGH (1 μg/ml, 24 h) and stained with an anti-TFAM monoclonal antibody (left). Equatorial-plane images from 200–300 cells were collected in random fields and analyzed using ImageJ software. Results are shown as mean ± SD after background-subtraction. Paired *t*-test *p<0.05, ****p<0.0001. *TFAM* expression levels (right) determined by RT-qPCR in untreated or rhGH-treated GM-MØ and M-MØ. Results are shown as in **(B)**. Paired *t*-test *p<0.05. **(F)** Mitochondrial DNA content, assessed by *MT-RNR2*, *MT-CO2* and *MT-ATP6* DNA abundance, determined by qPCR in GM-MØ and M-MØ untreated or treated with rhGH (1 μg/ml, 24 h). Average results of technical triplicates (n=8) are shown as 2^−ΔΔCt^ relative the mean nuclear DNA content as determined by the averaged levels of *APP* and *B2M* single-exon regions; box and whisker plots represent the median, second and third quartiles, and the minimum to maximum values. Paired *t*-test *p<0.05, **p<0.01.

In addition to their key bioenergetics role, mitochondria also produce metabolic precursors for macromolecules such as lipids, proteins and nucleic acids, and they also generate metabolic by-products such as ROS and ammonia (37). As mitochondria are dynamic and fuse and divide according to the metabolic and physiological needs of the cell, we hypothesized that GH might affect these processes in GM-MØ. We stained GM-MØ and M-MØ, untreated or GH-treated with MitoTracker Green (used to assess mitochondria number) for microscopy analysis. We found that the mitochondrial number was significantly higher in GM-MØ than in M-MØ, and that GH treatment significantly decreased the mitochondrial number in GM-MØ (**Figure 3A**; **Supplementary Figure 1C**).

Mitochondria abundance is regulated through biogenesis, fusion/fission events, and mitophagy. A key protein for mitochondrial biogenesis is peroxisome proliferator-activated receptor gamma coactivator 1α (PPARGC1α), whose activation triggers the subsequent activation of transcriptional regulators including nuclear respiratory factors (NRF1 and 2) and peroxisome proliferator-activated receptors (PPARs) (38), which initiate transcription of nuclear genes involved in mitochondrial biogenesis and function. RT-qPCR analysis of untreated and GH-treated GM-MØ and M-MØ showed that relative *PPARGC1α* expression was significantly higher in M-MØ than in GM-MØ, and that GH treatment significantly upregulated its expression in GM-MØ but not in M-MØ (**Figure 3B**). Moreover, a quantitative analysis of the immunofluorescence images confirmed the upregulation of PPARGC1α and demonstrated a GH-mediated nuclear translocation of PPARGC1α in GM-MØ, supporting its activation status (**Figures 3C, D**). A similar result was found for the transcription factor TFAM, which regulates mitochondrial biogenesis, both at the mRNA and protein levels (**Figure 3E**; **Supplementary Figure 1C**). TFAM is abundantly expressed in human mitochondria, as it coats the entire mitochondrial DNA (mtDNA), and serves essential roles in mtDNA transcription (39), replication (40), maintenance (41), packaging (42), and in nucleoid formation. Supporting this result, PCR analysis revealed a significant increase in the abundance of mtDNA in GH-treated GM-MØ but not in GH-treated M-MØ, as assessed by the quantification of *MT-CO2*, *MT-RNR2* and *MT-ATP6* genes DNA (**Figure 3F**), confirming the positive effect of GH on mtDNA levels in GM-MØ.

**Figure 4:**
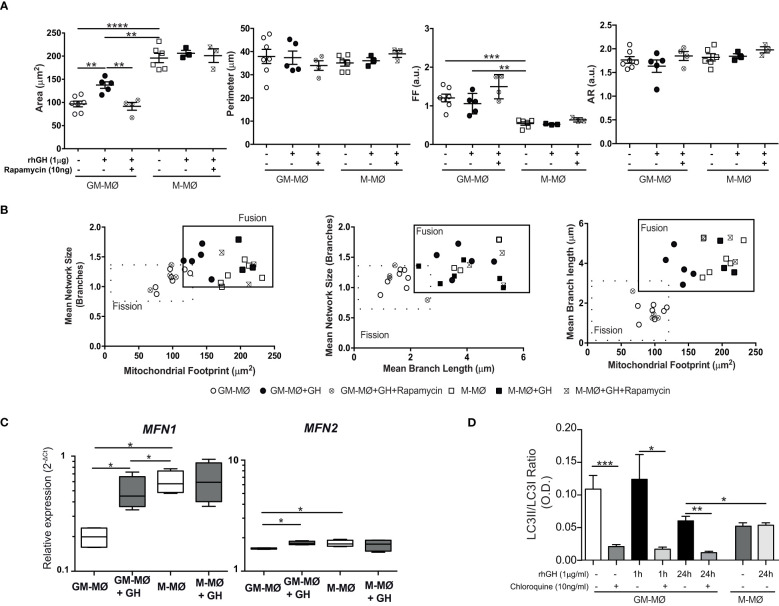
rhGH modulates mitochondrial dynamics in GM-MØ **(A)**. Quantitative analysis using specific macro tools of ImageJ of the area, perimeter, Form Factor **(FF)** and Aspect Ratio (AR) of untreated or rhGH-treated GM-MØ and M-MØ that were or not pretreated with rapamycin, as indicated. The y-axis in each dot plot represents the feature values of individual cells (n≥8). Line and error bars represent mean and standard error of mean. Paired *t*-test **p<0.01, ***p<0.001, ****p<0.0001. **(B)** Two-dimensional scatter plots from mitochondrial network analysis (footprints, network size, and branch length) matching data of individual cells plotted in **(A)**. The quadrants of fission/fusion were added to enclose clustered macrophages in feature space based on their mitochondrial organization. Dots are data of individual cells (n≥8). All data shown are representative of three independent experiments. **(C)** *MFN1* and *MFN2* mRNA expression levels determined by RT-qPCR in GM-MØ and M-MØ untreated or treated with rhGH (1 μg/ml, 24 h). Average results of technical triplicates (n=5) are shown as 2^−ΔCt^ relative to the mean of internal *TBP* expression; box and whisker plots represent the median, second and third quartiles, and the minimum to maximum values. Paired t-test *p<0.05. **(D)** Densitometric analysis of western blot analysis for LC-3I and LC-3II expression in lysates of GM-MØ and M-MØ untreated or treated with rhGH (1 μg/ml, 24 h) at different time points. A control for the effect of chloroquine (10 ng/ml, 30 min) pretreatment on untreated or rhGH-treated GM-MØ is also shown. Data were normalized to those of vinculin and expressed as a LC3II/LC3I ratio. Mean ± SD (n=4). Paired *t*-test *p<0.05, **p<0.01, ***p<0.001.

To explain the apparent discrepancy between the reduction in mitochondria numbers (assessed by dye staining) and biogenesis upregulation, we investigated the effect of GH on mitochondria dynamics by evaluating fusion and fission processes. Untreated and GH-treated GM-MØ and untreated M-MØ (used as a control) were fixed and stained with a monoclonal antibody to the mitochondrial membrane protein TOM22 (31). In agreement with previous results in LPS/IFN-activated macrophages (43), we observed that mitochondria (and mean branch length) were shorter in GM-MØ than in M-MØ (fission profile), which correlates with their glycolytic profile. Conversely, the elongated mitochondria in M-MØ (fusion profile) concurred with their previously described higher OCR/ECAR ratio (35). Notably, GH treatment of GM-MØ decreased fission and increased fusion (**Figures 4A, B**; **Supplementary Figure 2C**), in agreement with an effect of GH on reprogramming the metabolic profile of inflammatory macrophages (44). By contrast, GH failed to modify these processes in M-MØ (**Figures 4A, B**). The effect of GH on GM-MØ was abrogated by rapamycin (**Figures 4A, B**; **Supplementary Figure 2C**), confirming the involvement of the mTOR pathway in the effect of GH on GM-MØ. Mitochondria fusion events involve mitofusins on the outer membrane. We noted that the relative expression of mitofusin *MFN1* and *MFN2* mRNAs was higher in M-MØ than in GM-MØ, and that GH treatment upregulated their expression in GM-MØ (**Figure 4C**). The results were similar when MFN2 staining was evaluated (**Supplementary Figures 1C**, **4A**). Moreover, GH treatment of GM-MØ upregulated optic atrophy 1 (OPA-1) (45), a key mediator in fusion events (**Supplementary Figure 4B**). These data confirm a role for GH in enhancing mitochondrial fusion events in GM-MØ.

In addition to mitochondrial biogenesis and dynamics, mitophagy is also essential to maintain overall mitochondrial homeostasis by selectively removing aged and damaged mitochondria *via* the specific sequestration and engulfment of mitochondria for subsequent lysosomal degradation in a process promoted by M2-inducer factors such as CCL2 or IL-6 (46). LC3-II is a protein marker reliably associated with the formation of the autophagosome (47) and, as expected, western blotting analysis revealed a higher LC3II/LC3I ratio in GM-MØ (inflammatory macrophages) than in M-MØ. We also found that GH treatment of GM-MØ led to the time-dependent accumulation of LC3-I (**Figure 4D**; **Supplementary Figure 4C**). As a control for this analysis, the LC3-II/I ratio in GM-MØ was reduced by incubation with chloroquine, a drug that inhibits the progression of autophagic degradation in lysosomes with the subsequent accumulation of LC3I (48). These data confirm that LC3-mediated mitophagy events are favored in inflammatory over anti-inflammatory macrophages and suggest that GH treatment of GM-MØ likely abrogates this process. Overall, our findings indicate that GH might regulate the efficient production of ATP and the production of lipids and proteins in GM-MØ by influencing the number, structure and dynamics of mitochondria.

**Figure 5:**
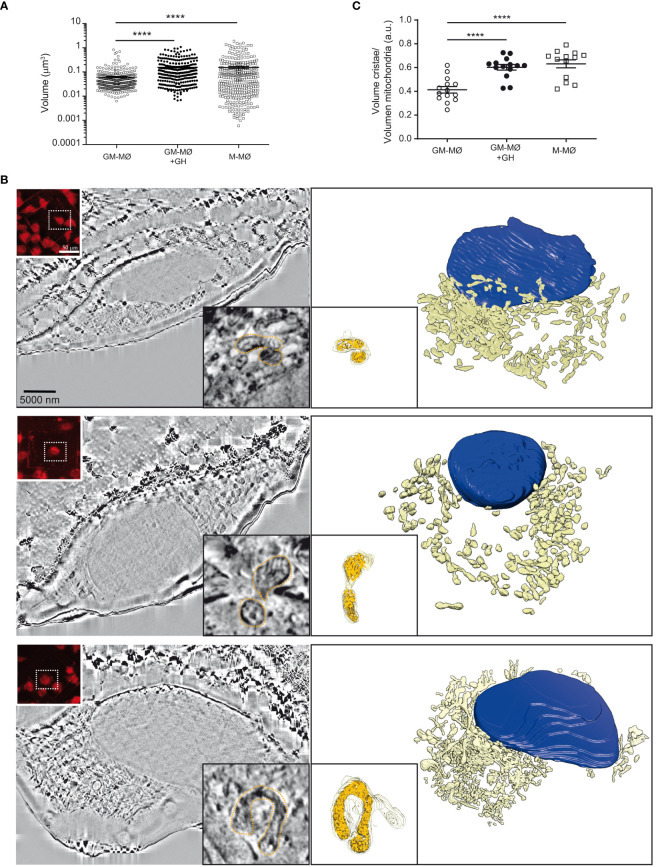
rhGH alters the mitochondria intimal structure of GM-MØ. **(A)** Mitochondrial volume from each experimental condition, untreated or rhGH-treated GM-MØ and M-MØ. Line and error bars represent mean and SEM. Paired *t*-test ****p<0.0001. **(B)** Reconstruction of untreated GM-MØ (upper), rhGH-treated GM-MØ (middle) and untreated M-MØ (lower). Scale bar: 5000 nm. In all cases, a representative cryo-FIB-SEM 3D section is shown (left) with a confocal microscopy image of the MitoTracker signal (treated 30 min at 37°C) (upper left corner inset. Scale bar: 50 μm), and a detailed image of one of the layers of the mitochondrion used to quantitate the volumes (right lower corner inset. Scale bar: 1 nm). Figure also shows the corresponding 3D segmentation (right) with a 3D representation of a mitochondrion (lower left corner inset) surface (yellow) and its inner structure (orange). **(C)** Quantification of the mitochondrial cristae volume relative to the total volume of the mitochondria corresponding to untreated GM-MØ, rhGH-treated GM-MØ and untreated M-MØ. Three representative mitochondria from each treatment per cell were analyzed (4 cells per donor, 2 different donors). Line and error bars represent mean and standard error of mean. Paired *t-*test ****p<0.0001.

Finally, we combined confocal live-cell imaging with correlative cryogenic fluorescence microscopy and cryo-FIB-SEM volume imaging to analyze the effect of GH on the shape and 3D-organization of mitochondria and their cristae. GM-MØ, M-MØ and GH-treated GM-MØ were seeded on finder gold grids and then stained with MitoTracker Red FM (a mitochondrial membrane-dependent dye) prior to sample vitrification and evaluation by cryo-fluorescence microscopy. Analysis indicated that total mitochondrial volume, and presumably mitochondrial mass, was smaller in GM-MØ than in M-MØ, and that GH treatment of GM-MØ significantly increased mitochondrial volume (**Figure 5A**). Additionally, we confirmed that GM-MØ mitochondria harbored a fission profile, whereas M-MØ mitochondria harbored a fusion profile, and that GH treatment of GM-MØ reduced fission and increased fusion events (**Supplementary Figures 5A, B**), an effect that was also evident when the elongation coefficient of the mitochondria was determined. Indeed, elongation was significantly higher in M-MØ and in GH-treated GM-MØ than in untreated GM-MØ (**Supplementary Figure 5C**). We also performed a quantitative analysis of the mitochondrial ultrastructure by determining the ratio between the cristae volume and the mitochondria volume as a measurement of the density and/or volume of cristae per mitochondria. Results indicated a higher density of cristae in anti-inflammatory (M-MØ) macrophages than in inflammatory (GM-MØ) macrophages, and GH increased the density of cristae in GM-MØ (**Figures 5B, C**). These data correlate well with the metabolic profile shift in GH-treated GM-MØ.

## Discussion

Macrophages comprise a functionally heterogeneous cell population with vital roles in host defense against pathogenic infections and in inflammatory responses. Their extreme phenotypic plasticity allows them to shape their responses to multiple microenvironmental cues. Classically-activated (M1) macrophages are potent effector cells that kill microorganisms and secrete inflammatory factors, whereas alternatively-activated (M2) macrophages scavenge debris, promote allergy reactions, participate in tissue remodeling and repair, and secrete anti-inflammatory cytokines (49). Both types use distinct pathways for energy production: whereas LPS/IFN-activated macrophages have enhanced glucose consumption and lactate release, IL-4-activated macrophages mainly use oxidative glucose metabolism (50, 51). Metabolic requirements influence macrophage plasticity, and their metabolic reprogramming regulates how they execute specific effector functions. Accordingly, instructing macrophage metabolism is a potential strategy to modulate their activity, which might be useful in diseases with a high macrophage commitment (52, 53). For example, by promoting the polarization of inflammatory macrophages in atheroma plaques towards an anti-inflammatory phenotype, IL-13 reduces atherosclerosis (54). Similarly, decreasing lipid levels or enhancing HDL levels switches inflammatory macrophages to an anti-inflammatory phenotype in atheroma plaques and triggers atherosclerosis regression (55).

While GM-CSF- and M-CSF-derived macrophages require LPS/IFN or IL-4/IL-10 activation, respectively, to generate classically- or alternatively-activated macrophages, analysis of their gene expression profile indicates that GM-CSF and M-CSF prime macrophages towards M1 and M2 phenotypes and, therefore, they can be considered inflammatory or anti-inflammatory (56, 57). We recently reported that GH reprograms GM-MØ to an anti-inflammatory and reparative phenotype (24). Supporting this, GH treatment of GM-MØ promoted a significant enrichment of anti-inflammatory gene expression while reducing the expression of genes linked to inflammation. GH also dampened the pro-inflammatory cytokine profile and upregulated IL-10 production, in agreement with the improved remission of inflammation and mucosal repair during recovery in the acute dextran sodium sulfate (DSS)-induced colitis model in bovine GH-overexpressing mice (24). Given this background, we hypothesized that GH treatment reprograms human GM-MØ metabolism, relatively suppressing glycolysis, lactate accumulation and ROS production. Previous data (35) support this hypothesis, and protein expression analysis revealed a GH-mediated downregulation of PKM2 and LDHA, two key proteins involved in glycolysis. PKM2 is an important regulator of HMGB1 secretion, and this danger/damage-associated molecular pattern, secreted together with TNFα, has been associated with inflammatory macrophages and can cause lethal sepsis (30, 58). Direct TNFα suppression partially inhibits LPS-induced HMGB1 release in macrophages, suggesting that HMGB1 secretion is partly mediated by a TNFα-dependent mechanism (59). We previously showed that GH treatment of GM-MØ inhibits TNFα production (24) and downregulates PDK1, an enzyme that plays a pivotal role in glucose metabolism. Knockdown of PDK1 blunts M1 activation but enhances M2 activation (60). In contrast to its effects on GM-MØ metabolism, we failed to observe any effect of GH on M-MØ metabolism. Confirming its role in suppressing glycolysis in GM-MØ, GH treatment reduced the mRNA levels of the glucose transporter *GLUT1*. GLUT1 is the primary rate-limiting glucose transporter in pro-inflammatory-polarized macrophages and its rapid upregulation is associated with a pro-inflammatory phenotype (2). This observation correlates with published data showing that human GH reduces the synthesis of GLUT1 and inhibits 2-deoxyglucose and 3-O-methyl-D-glucose uptake in 3T3-F442A adipose cells (61).

Consistent with the decrease in LDHA expression by GH, we also detected a decline in lactate accumulation in GM-MØ. Lactate has a critical function in inducing M2-like polarization in tumor models (62). In LPS/IFN-activated macrophages, the generation of lactate *via* pyruvate is essential to restore NAD^+^ levels and maintain flux through the glycolytic pathway (63). We speculate that the observed drop in lactate levels after GH treatment of GM-MØ correlates with the hormone-triggered downregulation of glycolysis and ROS production. These observations accord with a GH-mediated change in the bioenergetic profile of inflammatory macrophages. We also detected that GH treatment of GM-MØ increased mRNA levels of *CS*, encoding the enzyme involved in citrate production, whereas ACLY and *SLC25A1* expression were decreased. SLC25A1 is involved in citrate transport from mitochondria to the cytoplasm where ACLY catalyzes acetyl-CoA formation for *de novo* lipogenesis and histone acetylation reactions (64). ACLY has been associated with activated inflammatory macrophages and is related to the presence of human atherosclerotic plaques. Indeed, ACLY deficiency in myeloid cells induces a stable plaque phenotype, as demonstrated by increased collagen content and fibrous cap thickness, along with a smaller necrotic core size (65). In humans, severe GH deficiency is associated with increased cardiovascular risk and intima-media thickness at major arteries, a phenotype that can be reversed with rhGH replacement therapy (66).

A previous report (35) indicated that the levels of TCA metabolites were significantly higher in GM-MØ than in M-MØ, in agreement with our results, and suggesting that GH downregulates the levels of these metabolites in GM-MØ. We also detected lower levels of intracellular itaconate and of *ACOD1*, which encodes the enzyme that converts *cis*-aconitate to itaconate, in GH-treated inflammatory macrophages. Itaconate accumulation is a prime indicator of metabolic reprogramming in macrophages upon LPS treatment (67) and it is consistently detected in inflammatory macrophages where it is known to play an immunomodulatory role (26) by promoting anti-inflammatory functions (68). Using a murine transgenic model of DSS-induced colitis, we previously detected a higher proportion of Arginase^+^ cells infiltrating the colon of GHTg mice compared with wild-type littermates, which had increased number of iNOS^+^ cells (24). Flow cytometry analysis of these infiltrated macrophages in the intestinal tissues revealed a higher percentage of anti-inflammatory macrophages (CD45^+^F4/80^+^Gr1^+^CD11b^+^CD86^high^) in GHTg mice compared with controls (24). These data link the *in vitro* effects triggered by GH on macrophage metabolism with *in vivo* consequences, indicating that the effect also occurs in conditions in which the commitment of GM-MØ is conditioned by the surrounding environmental input.

Mitochondria are signaling organelles that regulate a wide variety of cellular functions and can dictate cell fate. Their structure is highly dynamic and their organization can determine the metabolic function of cells (69). Several controversial observations have been published relating mitochondrial mass to inflammation. For example, it was shown that an increase in mitochondrial mass is necessary for pro-inflammatory differentiation of macrophages (70), and that mitochondrial mass may be critical for the control of cell fate and immune responses (71). Also, higher mitochondrial mass was shown to contribute to T-cell senescence and cancer cell chemo-resistance (72, 73), whereas PGC-1α-deficient mice showed a lower mitochondrial mass, which causes spontaneous kidney inflammation and injury (74), and LPS treatment increased mitochondrial mass in macrophages together with pro-inflammatory cytokine production (70). We found that the mitochondria number was significantly lower in GH-treated GM-MØ than in untreated cells; however, their volume and area were upregulated, suggesting a GH-mediated increase of mitochondrial mass. Mitochondria continuously remodel their structure through biogenesis and mitophagy, and through alternate processes of fission and fusion. Correlating with the GH-mediated increase of mitochondrial mass, our results indicated that GH treatment stimulates mitochondria biogenesis, reduces fission and increases fusion events and reduces LC3-mediated mitophagy in inflammatory GM-MØ. We also detected increased mitofusin mRNA levels and MFN2 staining in GH-treated GM-MØ. Mitofusins are required on the outer membrane of mitochondria to allow fusion processes (75). These data correlated with GH-mediated upregulation of OPA-1 in these cells, an essential GTPase responsible for fusion of the mitochondrial inner membrane (76). Fusion events are associated with increased ATP production, activated OxPhos and ROS production (77). Mitochondrial fusion is also required to maintain the stoichiometry of the mtDNA replisome and the integrity of the mitochondrial genome (78). We also found an upregulation of TFAM in rhGH-treated GM-MØ. TFAM is a multifunctional DNA-binding protein that is essential for transcriptional activation and mtDNA organization (79). Mice lacking TFAM have impaired mtDNA transcription and are unable to maintain mtDNA, resulting in bioenergetic failure and embryonic lethality (80). GH-mediated TFAM upregulation correlated with the increased expression levels of mtDNA.

Analysis of mitochondria shape and internal structure using correlative cryogenic fluorescence microscopy combined with cryo FIB-SEM indicated a GH-mediated increase of GM-MØ mitochondria volume and elongation, which concurred with the increase in fusion events. We also detected a higher density of mitochondrial cristae in GM-MØ treated with GH, in agreement with a metabolic shift in inflammatory macrophages.

Signaling through mTOR is the most important intracellular pathway co-ordinating cellular metabolism (81), as it stimulates glycolysis and glucose uptake through the transcription factor hypoxia-inducible factor-1 alpha (82). In particular, the PI3K/Akt/mTOR signaling pathway is essential for macrophage activation, controlling either canonical signaling (e.g., JNK, NF-kB) or metabolic processes (83). We observed that the GH-mediated effects on mitochondria dynamics were abrogated in rapamycin-treated GM-MØ, in agreement with our previous results showing that GH-mediated reprogramming of GM-MØ is an AKT-mediated process (24), and with reports demonstrating that mTORC1 (mTOR complex1) is downstream of AKT (84).

Although we have observed *in vivo* that GH improves remission of inflammation and mucosal repair during recovery in a colitis model (24), our finding supporting a role for GH in reprogramming GM-MØ metabolism is based on *in vitro* assays, and therefore might be considered reductionist, as monocyte/macrophage activity and their functional commitment are strongly conditioned by the surrounding environmental and by metabolic input. Evaluation of the impact of GH treatment on primary monocytes isolated from patients experiencing inflammatory conditions, including atherosclerosis, colitis, obesity and even aging might contribute to strengthen the clinical relevance of our observations.

In conclusion, our results support a modulatory function of GH in the metabolism of the inflammatory macrophages that might contribute to immune regulation directly or indirectly through metabolic coupling between macrophages and other cell types. As macrophage metabolism is inextricably linked to their functionality, metabolic reprogramming might be an elegant way to intervene in multiple inflammatory diseases such as atherosclerosis or adipose tissue obesity-induced insulin resistance (85). We have recently shown that, *in vitro*, GH increases the phagocytic capacity of GM-MØ, a cell function classically associated with M-MØ, and reduces their antigen-presenting capacity (24). Epidemiological evidence suggests that aging is the single biggest risk factor for chronic inflammatory diseases and, mechanistically, inflammation is thought to be a common link between aging and disease. The metabolism of macrophages and, in particular, their metabolic repolarization, thus offers new therapeutic opportunities to treat inflammatory diseases and cancer.

## Data availability statement

The datasets presented in this study can be found in online repositories. The names of the repository/repositories and accession number(s) can be found in the article/**Supplementary Material**.

## Ethics statement

The studies involving human participants were reviewed and approved by Comité de ética del CSIC. The patients/participants provided their written informed consent to participate in this study.

## Author contributions

BS, RV, JR-F, and MM conceptualized the project and designed all aspects of the study. BS, PL and RV performed experiments. AC and CS developed the algorithms required for imaging analysis. BS, JP, DD and JC performed the correlative cryogenic fluorescence microscopy and cryo FIB-SEM. ML and FC quantitated itaconate levels. BS, MF and CB developed the metabolomic analysis. CR, NM, FI, JR-F, RV and MM provided input into the project. BS, RV and MM wrote the manuscript. All authors contributed to the article and approved the submitted version.

## Supplementary material

Supplementary Video 1 Example workflow of the followed cryo-correlative method applied to GM-macrophages growing on a TEM grid.

Supplementary Figure 1 Immunostaining images from untreated or rhGH-treated GM-MØ and M-MØ. **(A)** Calibration curve for the determination of itaconic acid concentration by UV detection. **(B)** GSEA on the ‘log fold-change-ranked’ list of genes obtained from untreated versus rhGH-treated GM-MØ according to limma analysis, using the previously defined MTORC1 genes set (Hallmark_MTORC1_SIGNALING; https://www.gsea-msigdb.org/gsea/msigdb/cards/HALLMARK_MTORC1_SIGNALING.html). Vertical black lines indicate the position of each of the genes comprising the glycolysis gene-set. The normalized enrichment score (NES), nominal p value and False Discovery Rate-adjusted Q value (FDRq) are presented. **(C)** Representative CMXRos, MitoTracker, MitoROS, PKM2, LDHA, ACLY, IDH, SDH, TFAM and MFN2 immunostaining images, as indicated, from untreated GM-MØ, rhGH-treated GM-MØ and untreated M-MØ. Scale bar: 50 μm. Images shown were obtained by confocal microscopy and used to obtain the data shown in **Figures 1B, 1G**, **2C, 2G**, **3A** and **Supplementary Figure 4A**. Selected images are shown (n=8). **(C)** ROS levels in untreated or rhGH-treated GM-MØ, and in M-MØ cells, was determined using DCFH-DA and flow cytometry. Representative plots are shown (n=8).

Supplementary Figure 2 Workflow of 2D images. **(A)**. Workflow of 2D confocal microscopy images for the analysis of anti-TOM22-labeled samples. **(B)**. Representative images of GM-MØ and M-MØ untreated or treated with rhGH (1 μg/ml, 24 h) and stained with an anti-TOM22 monoclonal antibody. (C) Quantitative analysis of branches and branch length of mitochondria from cells as in (5A). Dots are data from individual cells (n≥8) of three independent experiments performed. Results are mean ± SD. Paired *t*-test **p<0.01, ***p<0.001, ****p<0.0001.

Supplementary Figure 3 Workflow of Cryo-FIB-SEM of a macrophages grown on a grid. **(A)**. Cryo-confocal overlayed image of bright field and MitoTracker Red FM signal on a representative grid. Scale bar: 250 µm. **(B)**. SEM image of the same sample shown in A. **(C)**. Overlaying of cryo-fluorescence MitoTracker signal with SEM image shown in B. **(D)**. On the left, SEM image of the macrophage chosen for analysis based on correlative information shown in C and on the right, same area highlighted (black square) in the correlated image. Scale bar: 250 μm. **(E)**. A slice through a macrophage whole FIB-SEM volume after image processing. Scale bar: 5000 nm. **(F)**. Three-dimensional rendering of the segmentation performed in the FIB-SEM volume shown in E (nucleus, blue; mitochondria, light yellow).

Supplementary Figure 4 Treatment of GM-MØ with rhGH increases mitochondria fusion and reduces mitophagy. **(A)** Quantitative analysis of confocal images of GM-MØ and M-MØ untreated or treated with rhGH (1 μg/ml, 24 h) stained with an anti-MFN2 monoclonal antibody. Equatorial-plane images from 200–300 cells were collected in random fields and analyzed using ImageJ software. Results are shown as mean ± SD after background-subtraction. Paired *t-*test ****p<0.0001. **(B)** GM-MØ and M-MØ untreated or treated with rhGH (1 μg/ml, 24 h) were lysed and the expression of OPA1 was determined by western blotting using an anti-OPA1 monoclonal antibody. As loading control membranes were reblotted with an anti-vinculin monoclonal antibody. A representative experiment of 5 performed is shown. Densitometric analysis of the western blotting performed is also shown. Results were normalized to that of vinculin, and shown as mean ± SEM (n=5). Paired *t*-test, *p<0.05. **(C)** Representative western blotting analysis of LC-3I and LC-3II expression in lysates of GM-MØ and M-MØ untreated or rhGH-treated at different time points. A control for the effect of chloroquine (10 ng/ml, 30 min) pretreatment on untreated or rhGH-treated GM-MØ is also shown. For equally loading control membrane was reblotted with anti-vinculin monoclonal antibody.

Supplementary Figure 5 Treatment of GM-MØ with rhGH modulates mitochondrial ultrastructure. **(A)** Two-dimension al scatter plots from mitochondrial network analysis (volume, network size, and branch length) comparing untreated or rhGH-treated GM-MØ, and M-MØ. The quadrants of fission/fusion were added to enclose clustered macrophages in feature space based on their mitochondrial organization. Dots are data of individual cells (n=3). All data shown are representative of three independent runs of experiments. **(B)** Quantitative analysis of network size and branch length of mitochondria from untreated or rhGH-treated GM-MØ, and M-MØ. Dots are data from individual cells (n=3) of three independent experiments. Results are expressed as mean ± SD. Paired *t*-test *p<0.05, **p<0.01, ****p<0.0001. **(C)** Quantification of the elongation of untreated or rhGH-treated GM-MØ and M-MØ. Results are expressed as mean ± SD. Paired *t*-test *p<0.05.

## References

[1] Gordon S Taylor PR. Monocyte and macrophage heterogeneity. Nat Rev Immunol (2005) 5(12):953–64. doi: 10.1038/nri1733 16322748

[2] Freemerman AJ Johnson AR Sacks GN Milner JJ Kirk EL Troester MA. Metabolic reprogramming of macrophages: glucose transporter 1 (GLUT1)-mediated glucose metabolism drives a proinflammatory phenotype. J Biol Chem (2014) 289(11):7884–96. doi: 10.1074/jbc.M113.522037 PMC3953299 24492615

[3] Jha AK Huang SCC Sergushichev A Lampropoulou V Ivanova Y Loginicheva E. Network integration of parallel metabolic and transcriptional data reveals metabolic modules that regulate macrophage polarization. Immunity (2015) 42(3):419–30. doi: 10.1016/j.immuni.2015.02.005 25786174

[4] Feingold KR Shigenaga JK Kazemi MR McDonald CM Patzek SM Cross AS. Mechanisms of triglyceride accumulation in activated macrophages. J Leukocyte Biol (2012) 92(4):829–39. doi: 10.1189/jlb.1111537 PMC3441312 22753953

[5] Vats D Mukundan L Odegaard JI Zhang L Smith KL Morel CR. Oxidative metabolism and PGC-1β attenuate macrophage-mediated inflammation. Cell Metab (2006) 4(1):13–24. doi: 10.1016/j.cmet.2006.05.011 16814729 PMC1904486

[6] Haschemi A Kosma P Gille L Evans CR Burant CF Starkl P. The sedoheptulose kinase CARKL directs macrophage polarization through control of glucose metabolism. Cell Metab (2012) 15(6):813–26. doi: 10.1016/j.cmet.2012.04.023 PMC3370649 22682222

[7] De Santa F Vitiello L Torcinaro A Ferraro E. The role of metabolic remodeling in macrophage polarization and its effect on skeletal muscle regeneration. In Antioxidants Redox Signaling (2019) 30:1553–98. doi: 10.1089/ars.2017.7420 30070144

[8] Woods PS Kimmig LM Meliton AY Sun KA Tian Y O’Leary EM. Tissue-resident alveolar macrophages do not rely on glycolysis for LPS-induced inflammation. Am J Respir Cell Mol Biol (2020) 62(2):243–55. doi: 10.1165/rcmb.2019-0244OC PMC6993551 31469581

[9] Puthenveetil A Dubey S. Metabolic reprograming of tumor-associated macrophages. Ann Trans Med (2020) 8(16):1030–0. doi: 10.21037/atm-20-2037 PMC7475460 32953830

[10] Wenes M Shang M Di Matteo M Goveia J Martín-Pérez R Serneels J. Macrophage metabolism controls tumor blood vessel morphogenesis and metastasis. Cell Metab (2016) 24(5):701–15. doi: 10.1016/j.cmet.2016.09.008 27773694

[11] Ogle GD Rosenberg AR Kainer G. Renal effects of growth hormone. II. electrolyte homeostasis and body composition. Pediatr Nephrol (1992) 6(5):483–9. doi: 10.1007/BF00874021 1457334

[12] Veldhuis JD Roemmich JN Rogol AD. Gender and sexual maturation-dependent contrasts in the neuroregulation of growth hormone secretion in prepubertal and late adolescent males and females-a general clinical research center-based study*. J Clin Endocrinol Metab (2000) 85(7):2385–94. doi: 10.1210/jc.85.7.2385 10902783

[13] Velloso CP. Regulation of muscle mass by growth hormone and IGF-i. in. Br J Pharmacol (2008) 154(3):557–68. doi: 10.1038/bjp.2008.153 PMC2439518 18500379

[14] Kimata H Fujimoto M. Growth hormone and insulln-like growth factor I induce immunoglobulin (Ig)E and IgG4 production by human b cells. (1994) 180(2):727–32. doi: 10.1084/jem.180.2.727 PMC2191604 8046348

[15] Lu C Kumar PA Fan Y Sperling MA Menon RK. A novel effect of growth hormone on macrophage modulates macrophage-dependent adipocyte differentiation. Endocrinology (2010) 151(5):2189–99. doi: 10.1210/en.2009-1194 PMC2869256 20185763

[16] Murphy WJ Durum SK Longo DL. Human growth hormone promotes engraftment of murine or human T cells in severe combined immunodeficient mice. Proc Natl Acad Sci USA (1992) 89(10):4481–5. doi: 10.1073/pnas.89.10.4481 PMC49106 1584780

[17] Welniak LA Sun R Murphy WJ. The role of growth hormone in T-cell development and reconstitution. J Leukocyte Biol (2002) 71(3):381–7. doi: 10.1189/jlb.71.3.381 11867675

[18] Villares R Criado G Juarranz Y Lopez-Santalla M García-Cuesta EM Rodríguez-Frade JM. Inhibitory role of growth hormone in the induction and progression phases of collagen-induced arthritis. Front Immunol (2018) 9:1165(May). doi: 10.3389/fimmu.2018.01165 29887869 PMC5980961

[19] Villares R Kakabadse D Juarranz Y Gomariz RP Martínez-A C Mellado M. Growth hormone prevents the development of autoimmune diabetes. Proc Natl Acad Sci USA (2013) 110(48):E4619–27. doi: 10.1073/pnas.1314985110 PMC3845149 24218587

[20] Han X Benight N Osuntokun B Loesch K Frank SJ Denson LA. Tumour necrosis factor α blockade induces an anti-inflammatory growth hormone signalling pathway in experimental colitis. Gut (2007) 56(1):73–81. doi: 10.1136/gut.2006.094490 16777921 PMC1856672

[21] Slonim AE Bulone B Amore ABD Eresia T Oldberg G Ingertzahn AAW. A preliminary study of grow th hormone therapy for crohn’s disease. N Engl J Med (2000) 342(22):1633–7. doi: 10.1056/NEJM200006013422203 10833209

[22] Warwick-Davies J Lowrie DB Cole PJ. Growth hormone activation of human monocytes for superoxide production but not tumor necrosis factor production, cell adherence, or action against mycobacterium tuberculosis. Infect Immun (1995) 63(11):4312–1326. doi: 10.1128/iai.63.11.4312-4316.1995 7591064 PMC173613

[23] Smith JR Benghuzzi H Tucci M Puckett A Hughes JL. The effects of growth hormone and insulin-like growth factor on the proliferation rate and morphology of RAW 264.7 macrophages. Biomed Sci Instrum (2000) 36:111–6. 10834218

[24] Soler Palacios B Nieto C Fajardo P González de la Aleja A Andrés N Dominguez-Soto Á.. Growth hormone reprograms macrophages toward an anti-inflammatory and reparative profile in an MAFB-dependent manner. J Immunol (2020) 205(3):776–88. doi: 10.4049/jimmunol.1901330 32591394

[25] Spadaro O Goldberg EL Camell CD Youm YH Kopchick JJ Nguyen KY. Growth hormone receptor deficiency protects against age-related NLRP3 inflammasome activation and immune senescence. Cell Rep (2016) 14(7):1571–80. doi: 10.1016/j.celrep.2016.01.044 PMC5992590 26876170

[26] Hooftman A Angiari S Hester S Corcoran SE Runtsch MC Ling C. The immunomodulatory metabolite itaconate modifies NLRP3 and inhibits inflammasome activation. Cell Metab (2020) 32(3):468–78.e7. doi: 10.1016/j.cmet.2020.07.016 32791101 PMC7422798

[27] Liberzon A Birger C Thorvaldsdóttir H Ghandi M Mesirov JP Tamayo P. The molecular signatures database (MSigDB) hallmark gene set collection. Cell Syst (2015) 1(6):417–25. doi: 10.1016/j.cels.2015.12.004 PMC4707969 26771021

[28] Schindelin J Arganda-Carreras I Frise E Kaynig V Longair M Pietzsch T. Fiji: An open-source platform for biological-image analysis. Nat Methods (2012) 9(7):676–82. doi: 10.1038/nmeth.2019 PMC3855844 22743772

[29] Fiehn O. Metabolite profiling in Arabidopsis. Methods Mol Biol (2006) 323:439–47:439. doi: 10.1385/1-59745-003-0 16739598

[30] Kraja AT Liu C Fetterman JL Graff M Have CT Gu C. Associations of mitochondrial and nuclear mitochondrial variants and genes with seven metabolic traits. Am J Hum Genet (2019) 104(1):112–38. doi: 10.1016/j.ajhg.2018.12.001 PMC6323610 30595373

[31] Valente AJ Maddalena LA Robb EL Moradi F Stuart JA. A simple ImageJ macro tool for analyzing mitochondrial network morphology in mammalian cell culture. Acta Histochemica (2017) 119(3):315–26. doi: 10.1016/j.acthis.2017.03.001 28314612

[32] Lowe DG. Distinctive image features from scale-invariant keypoints. Int J Comput Vision (2004) 60(2):91–110. doi: 10.1023/b:visi.0000029664.99615.94

[33] Mootha VK Lindgren CM Eriksson K-F Subramanian A Sihag S Lehar J. PGC-1alpha-responsive genes involved in oxidative phosphorylation are coordinately downregulated in human diabetes. Nat Genet (2003) 34(3):267–73. doi: 10.1038/ng1180 12808457

[34] Subramanian A Tamayo P Mootha VK Mukherjee S Ebert BL Gillette MA. Gene set enrichment analysis: a knowledge-based approach for interpreting genome-wide expression profiles. Proc Natl Acad Sci USA (2005) 102(43):15545–50. doi: 10.1073/pnas.0506580102 PMC1239896 16199517

[35] Izquierdo E Cuevas VD Fernández-Arroyo S Riera-Borrull M Orta-Zavalza E Joven J. Reshaping of human macrophage polarization through modulation of glucose catabolic pathways. J Immunol (2015) 195(5):2442–51. doi: 10.4049/jimmunol.1403045 26209622

[36] Zhang W Su J Xu H Yu S Liu Y Zhang Y. Dicumarol inhibits PDK1 and targets multiple malignant behaviors of ovarian cancer cells. PLoS One (2017) 12(6):e0179672. doi: 10.1371/journal.pone.0179672 28617852 PMC5472302

[37] Spinelli JB Haigis MC. The multifaceted contributions of mitochondria to cellular metabolism. in. Nat Cell Biol (2018) 20(7):745–54. doi: 10.1038/s41556-018-0124-1 PMC6541229 29950572

[38] Scarpulla RC. Transcriptional paradigms in mammalian mitochondrial biogenesis and function. Physiol Rev (2008) 88(2):611–38. doi: 10.1152/physrev.00025.2007 18391175

[39] Maniura-Weber K Goffart S Garstka HL Montoya J Wiesner RJ. Transient overexpression of mitochondrial transcription factor a (TFAM) is sufficient to stimulate mitochondrial DNA transcription, but not sufficient to increase mtDNA copy number in cultured cells. Nucleic Acids Res (2004) 32(20):6015–27. doi: 10.1093/nar/gkh921 PMC534614 15547250

[40] Pohjoismäki JLO Goffart S Tyynismaa H Willcox S Ide T Kang D. Human heart mitochondrial DNA is organized in complex catenated networks containing abundant four-way junctions and replication forks. J Biol Chem (2009) 284(32):21446–57. doi: 10.1074/jbc.M109.016600 PMC2755869 19525233

[41] Kanki T Ohgaki K Gaspari M Gustafsson CM Fukuoh A Sasaki N. Architectural role of mitochondrial transcription factor a in maintenance of human mitochondrial DNA. Mol Cell Biol (2004) 24(22):9823–34. doi: 10.1128/mcb.24.22.9823-9834.2004 PMC525493 15509786

[42] Alam TI Kanki T Muta T Ukaji K Abe Y Nakayama H. Human mitochondrial DNA is packaged with TFAM. In Nucleic Acids Res (2003) 31:1640–5. doi: 10.1093/nar/gkg251 PMC152855 12626705

[43] Ramond E Jamet A Coureuil M Charbit A. Pivotal role of mitochondria in macrophage response to bacterial pathogens. Front Immunol (2019) 10:2461(Oct). doi: 10.3389/fimmu.2019.02461 31708919 PMC6819784

[44] Nagdas S Kashatus DF. The interplay between oncogenic signaling networks and mitochondrial dynamics. Antioxidants (2017) 6(2):33. doi: 10.3390/antiox6020033 28513539 PMC5488013

[45] Sessions DT Kim K-B Kashatus JA Churchill N Park K-S Mayo MW. Opa1 and Drp1 reciprocally regulate cristae morphology, ETC function, and NAD+ regeneration in KRas-mutant lung adenocarcinoma. Cell Rep (2022) 41(11):111818. doi: 10.1016/j.celrep.2022.111818 36516772 PMC10265649

[46] Roca H Varsos ZS Sud S Craig MJ Ying C Pienta KJ. CCL2 and interleukin-6 promote survival of human CD11b+ peripheral blood mononuclear cells and induce M2-type macrophage polarization. J Biol Chem (2009) 284(49):34342–54. doi: 10.1074/jbc.M109.042671 PMC2797202 19833726

[47] Yoshii SR Mizushima N. Monitoring and measuring autophagy. Int J Mol Sci (2017) 18(9):1865–77. doi: 10.3390/ijms18091865 PMC5618514 28846632

[48] Mizushima N Yoshimori T. How to interpret LC3 immunoblotting. Autophagy (2007) 3:542–5. doi: 10.4161/auto.4600 17611390

[49] Wynn TA Chawla A Pollard JW. Macrophage biology in development, homeostasis and disease. Nature (2013) 496(7446):445–55. doi: 10.1038/nature12034 PMC3725458 23619691

[50] Rodríguez-Prados J-C Través PG Cuenca J Rico D Aragonés J Martín-Sanz P. Substrate fate in activated macrophages: a comparison between innate, classic, and alternative activation. J Immunol (2010) 185(1):605–14. doi: 10.4049/jimmunol.0901698 20498354

[51] Odegaard JI Chawla A. Alternative macrophage activation and metabolism. Annu Rev Pathology: Mech Dis (2011) 6:275–97. doi: 10.1146/annurev-pathol-011110-130138 PMC3381938 21034223

[52] O’Neill LAJ Pearce EJ. Immunometabolism governs dendritic cell and macrophage function. J Exp Med (2016) 213(1):15–23. doi: 10.1084/jem.20151570 26694970 PMC4710204

[53] O’Neill LAJ Kishton RJ Rathmell J. A guide to immunometabolism for immunologists. in. Nat Rev Immunol (2016) 16(9):553–565). doi: 10.1038/nri.2016.70 27396447 PMC5001910

[54] Stöger JL Gijbels MJJ van der Velden S Manca M van der Loos CM Biessen EAL. Distribution of macrophage polarization markers in human atherosclerosis. Atherosclerosis (2012) 225(2):461–8. doi: 10.1016/j.atherosclerosis.2012.09.013 23078881

[55] Feig JE Rong JX Shamir R Sanson M Vengrenyuk Y Liu J. HDL promotes rapid atherosclerosis regression in mice and alters inflammatory properties of plaque monocyte-derived cells. Proc Natl Acad Sci USA (2011) 108(17):7166–71. doi: 10.1073/pnas.1016086108 PMC3084076 21482781

[56] Martinez FO Gordon S Locati M Mantovani A. Transcriptional profiling of the human monocyte-to-Macrophage differentiation and polarization: new molecules and patterns of gene expression. J Immunol (2006) 177(10):7303–11. doi: 10.4049/jimmunol.177.10.7303 17082649

[57] Lacey DC Achuthan A Fleetwood AJ Dinh H Roiniotis J Scholz GM. Defining GM-CSF– and Macrophage-CSF–dependent macrophage responses by *In vitro* models. J Immunol (2012) 188(11):5752–65. doi: 10.4049/jimmunol.1103426 22547697

[58] Yang L Xie M Yang M Yu Y Zhu S Hou W. PKM2 regulates the warburg effect and promotes HMGB1 release in sepsis. Nat Commun (2014) 5:4436. doi: 10.1038/ncomms5436 25019241 PMC4104986

[59] Chen G Li J Ochani M Rendon-Mitchell B Qiang X Susarla S. Bacterial endotoxin stimulates macrophages to release HMGB1 partly through CD14- and TNF-dependent mechanisms. J Leukocyte Biol (2004) 76(5):994–1001. doi: 10.1189/jlb.0404242 15331624

[60] Tan Z Xie N Cui H Moellering DR Abraham E Thannickal VJ. Pyruvate dehydrogenase kinase 1 participates in macrophage polarization *via* regulating glucose metabolism. J Immunol (2015) 194(12):6082–9. doi: 10.4049/jimmunol.1402469 PMC4458459 25964487

[61] Ku Tai P Liao J-F Chen EH Dietz J SchwartzQ J Carter-Sun C. Differential regulation of two glucose transporters by chronic growth hormone treatment of cultured 3T3-F442A adipose cells. J Biol Chem (1990) 265(35):21828–34. doi: 10.1016/s0021-9258(18)45814-0 2254335

[62] Colegio OR Chu NQ Szabo AL Chu T Rhebergen AM Jairam V. Functional polarization of tumour-associated macrophages by tumour-derived lactic acid. Nature (2014) 513(7519):559–63. doi: 10.1038/nature13490 PMC4301845 25043024

[63] Viola A Munari F Sánchez-Rodríguez R Scolaro T Castegna A. The metabolic signature of macrophage responses. Front Immunol (2019) 10:1462(July). doi: 10.3389/fimmu.2019.01462 31333642 PMC6618143

[64] Sabari BR Zhang D Allis CD Zhao Y. Metabolic regulation of gene expression through histone acylations. Nat Rev Mol Cell Biol (2017) 18(2):90–101. doi: 10.1038/nrm.2016.140 27924077 PMC5320945

[65] Baardman J Verberk SGS van der Velden S Gijbels MJJ van Roomen CPPA Sluimer JC. Macrophage ATP citrate lyase deficiency stabilizes atherosclerotic plaques. Nat Commun (2020) 11(1):6296–311. doi: 10.1038/s41467-020-20141-z PMC7722882 33293558

[66] Colao A Galderisi M Di Sarno A Pardo M Gaccione M D’Andrea M. Increased prevalence of tricuspid regurgitation in patients with prolactinomas chronically treated with cabergoline. J Clin Endocrinol Metab (2008) 93(10):3777–84. doi: 10.1210/jc.2007-1403 18682513

[67] Lee C Jenkins N Gilbert D N. Cloning and analysis of gene regulation of a novel LPS-inducible cDNA. Immunogenetics (1995) 41(5):263–70. doi: 10.1007/BF00172150 7721348

[68] Murphy MP O’Neill LAJ. Krebs Cycle reimagined: the emerging roles of succinate and itaconate as signal transducers. Cell (2018) 174(4):780–4. doi: 10.1016/j.cell.2018.07.030 30096309

[69] Archer SL. Mitochondrial dynamics [[/amp]]mdash; mitochondrial fission and fusion in human diseases. New Engl J Med (2013) 369(23):2236–51. doi: 10.1056/nejmra1215233 24304053

[70] Yu W Wang X Zhao J Liu R Liu J Wang Z. Stat2-Drp1 mediated mitochondrial mass increase is necessary for pro-inflammatory differentiation of macrophages. Redox Biol (2020) 37:101761. doi: 10.1016/j.redox.2020.101761 33080440 PMC7575803

[71] Angajala A Lim S Phillips JB Kim J-H Yates C You Z. Diverse roles of mitochondria in immune responses: novel insights into immuno-metabolism. Front Immunol (2018) 9:1605. doi: 10.3389/fimmu.2018.01605 30050539 PMC6052888

[72] Callender LA Carroll EC Bober EA Akbar AN Solito E Henson SM. Mitochondrial mass governs the extent of human T cell senescence. Aging Cell (2020) 19(2):e13067. doi: 10.1111/acel.13067 31788930 PMC6996952

[73] Davizon-Castillo P McMahon B Aguila S Bark D Ashworth K Allawzi A. TNF-a-driven inflammation and mitochondrial dysfunction define the platelet hyperreactivity of aging. Blood (2019) 134(9):727–40. doi: 10.1182/blood.2019000200 PMC6716075 31311815

[74] Fontecha-Barriuso M Martín-Sánchez D Martinez-Moreno JM Carrasco S Ruiz-Andrés O Monsalve M. PGC-1α deficiency causes spontaneous kidney inflammation and increases the severity of nephrotoxic AKI. J Pathol (2019) 249(1):65–78. doi: 10.1002/path.5282 30982966

[75] Santel A Fuller MT. Control of mitochondrial morphology by a human mitofusin. J Cell Sci (2001) 114(5):867–74. doi: 10.1242/jcs.114.5.867 11181170

[76] Ishihara N Fujita Y Oka T Mihara K. Regulation of mitochondrial morphology through proteolytic cleavage of OPA1. EMBO J (2006) 25(13):2966–77. doi: 10.1038/sj.emboj.7601184 PMC1500981 16778770

[77] Wai T Langer T. Mitochondrial dynamics and metabolic regulation. Trends Endocrinol Metab (2016) 27(2):105–17. doi: 10.1016/j.tem.2015.12.001 26754340

[78] Chapman J Ng YS Nicholls TJ. The maintenance of mitochondrial DNA integrity and dynamics by mitochondrial membranes. In Life (2020) 10:1–42). doi: 10.3390/life10090164 PMC7555930 32858900

[79] Bonawitz ND Clayton DA Shadel GS. Initiation and beyond: multiple functions of the human mitochondrial transcription machinery. In Mol Cell (2006) 24:813–825). doi: 10.1016/j.molcel.2006.11.024 17189185

[80] Larsson N-G Wang J Wilhelmsson H Oldfors A Rustin P Lewandoski M. Mitochondrial transcription factor a is necessary for mtDNA maintenance and embryogenesis in mice. Nat Genet (1998) 18(3):231–6. doi: 10.1038/ng0398-231 9500544

[81] Saxton RA Sabatini DM. mTOR signaling in growth, metabolism, and disease. Cell (2017) 168(6):960–76. doi: 10.1016/j.cell.2017.02.004 PMC5394987 28283069

[82] Düvel K Yecies JL Menon S Raman P Lipovsky AI Souza AL. Activation of a metabolic gene regulatory network downstream of mTOR complex 1. Mol Cell (2010) 39(2):171–83. doi: 10.1016/j.molcel.2010.06.022 PMC2946786 20670887

[83] Covarrubias AJ Aksoylar HI Horng T. Control of macrophage metabolism and activation by mTOR and akt signaling. In Semin Immunol (2015) 27:286–296). doi: 10.1016/j.smim.2015.08.001 PMC4682888 26360589

[84] Kim J Guan KL. mTOR as a central hub of nutrient signalling and cell growth. Nat Cell Biol (2019) 21(1):63–71. doi: 10.1038/s41556-018-0205-1 30602761

[85] Geeraerts X Bolli E Fendt SM Van Ginderachter JA. Macrophage metabolism as therapeutic target for cancer, atherosclerosis, and obesity. Front Immunol (2017) 8:289. doi: 10.3389/fimmu.2017.00289 28360914 PMC5350105

